# The Student’s Manual of Venereal Diseases

**Published:** 1901-07

**Authors:** 


THE STUDENT'S MANUAL OF VENEREAL DISEASES. By F. R STURGIS, M. D, sometime Clinical Professor of Venereal Diseases of the Medical Department of the University of the City of New York, etc. Duodecimo, pp. 216. Seventh edition, revised and in part rewritten by the author and Follen Cabot, M. D., Instructor in Genito-urinary and Venereal Diseases in the Cornell University Medical College. Philadelphia: P. Blakiston's Son & Co. 1901. [Price, $1.25.]

The seventh edition of this work, which has been revised and, as was the first edition, dedicated to the medical students of the United States, needs but little criticism. The three venereal diseases are considered, not exhaustively, but in a manner that brings out every important point regarding their diagnosis and treatment. Sturgis devotes but six or seven lines to the hypodermic medication in syphilis, saying on account of the pain and irritation produced, inunctions are far preferable. This statement must be contradicted, for these injections can be given with scarcely a moment's suffering, and as to results obtained, saying nothing regarding the nastiness of being smeared with grease, can in no way compare with those obtained by hypodermic medication.

Gonorrhea is treated of in a rational manner and it is pleasing to see no mention made of most of the new fangled remedies, for there is no doubt Cabot has tried many only to find as all do, sooner or later, that their value has been overestimated. In italics appears the statement "that many cases are continued by overtreatment," yet, the advice is given to explore the urethra with the sound or bulb, and to use the endoscope in subacute or chronic conditions and also, after the acute condition in the posterior urethra has subsided, silver in strong solutions are advised to be used with the Ultzmann syringe. This advice is dangerous, until a certain chronic condition is reached, for those who carry out the instructions given will find their case prolonged, if not made worse, all due to the fact that the patient has been overtreated; in fact, instead of soothing the condition it has been irritated.

Cabot gives a long dissertation on drugs for chordee and mentions that hot water is of value when used at night. In reality chordee should almost be omitted as a symptom in gonorrhea, but when it does occur hot water should be used four or five times a day, immersing the penis for at least five minutes each time.

The reviewer must disagree with the statement that the female urethra is the most common site of gonorrheal inflammation, for as compared with the cervix it ranks as one to twenty.

There is no apparent reason why the vulva should not receive the same treatment as is advised for the vagina, viz.: a 10 to 20 per cent. solution of silver, instead of as Cabot advises a 1-1000 solution.

One make-up of this volume seems to call for criticism, —its appearance. Punctuation has today reached a stage where even the most important sentence can be brought out, or given extra notice without being italicised, and a much more pleasing face is given the page. Although there are a few trivial points that do not entirely meet with the reviewer's opinion upon the subject, the work as a whole is so concise and up-to-date, and is cheerfullyrecommended to all. J.H.D.

